# Western North Pacific Tropical Cyclone Activity in 2018: A Season of Extremes

**DOI:** 10.1038/s41598-020-62632-5

**Published:** 2020-03-27

**Authors:** Si Gao, Langfeng Zhu, Wei Zhang, Xinyong Shen

**Affiliations:** 1 School of Atmospheric Sciences, and Guangdong Province Key Laboratory for Climate Change and Natural Disaster Studies, Sun Yat-sen University, Zhuhai, China; 2 Southern Marine Science and Engineering Guangdong Laboratory (Zhuhai), Zhuhai, China; 3 Key Laboratory of Meteorological Disaster, Ministry of Education, and Collaborative Innovation Center on Forecast and Evaluation of Meteorological Disaster, Nanjing University of Information Science and Technology, Nanjing, China; 4 IIHR-Hydroscience and Engineering, The University of Iowa, Iowa City, IA USA

**Keywords:** Climate sciences, Atmospheric science

## Abstract

The 2018 tropical cyclone (TC) season over the western North Pacific (WNP) underwent two extreme situations: 18 TCs observed during June–August (JJA) and ranked the second most active summer in the satellite era; only 5 TCs that occurred during September–October (SO), making it the most inactive period since the late 1970s. Here we attribute the two extreme situations based on observational analyses and numerical experiments. The extremely active TC activity and northward shift of TC genesis during JJA of 2018 can be attributed to the WNP anomalous low-level cyclone, which is due primarily to El Niño Modoki and secondarily to the positive phase of the Pacific Meridional Mode (PMM). Overall, the extremely inactive TC activity during SO of 2018 is due to the absence of TC formation over the South China Sea and Philippine Sea, which can be attributed to the *in-situ* anomalous low-level anticyclone associated with the positive phase of the Indian Ocean Dipole, although the positive PMM phase and El Niño Modoki still hold.

## Introduction

**Figure 1:**
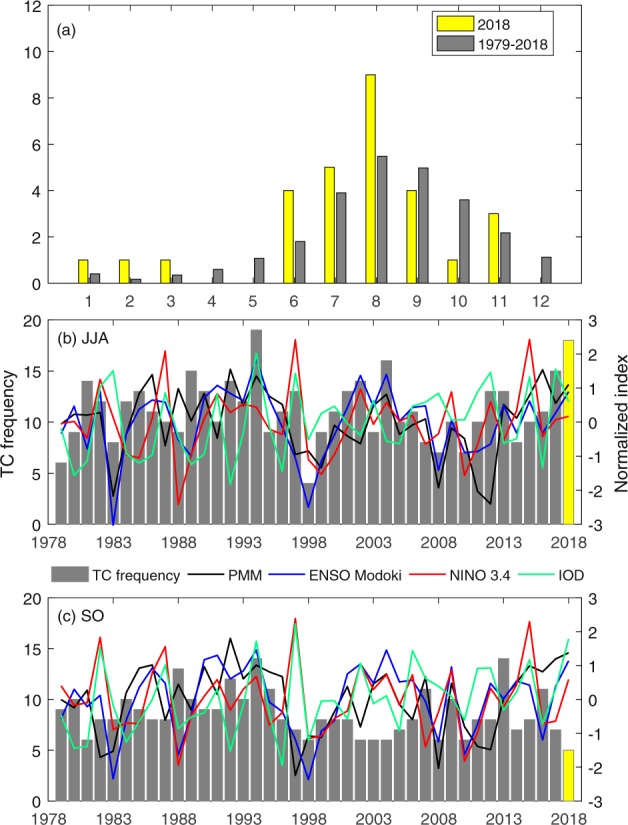
(**a**) Monthly TC genesis frequency in 2018 (yellow) compared with the 1979–2018 climatological mean (gray). Time series of TC genesis frequency (gray bars for 1979–2017 and yellow bar for 2018) and the PMM index (black line), ENSO Modoki index (blue line), IOD index (green line), and Niño 3.4 index (red line) during (**b**) JJA and (**c**) SO from 1979 to 2018 normalized by their respective standard deviations.

The western North Pacific (WNP) including the South China Sea (SCS) is the most active basin of tropical cyclone (TC) activity. On average, 20 TCs are observed over the WNP during the peak season (June to October, JJASO), with 11 TCs during June–August (JJA) and 9 TCs during September–October (SO). However, the 2018 WNP TC season was featured by a season of extreme cases (Fig. 1a): there were 18 TCs during JJA ranked the second largest number since 1979, whereas only 5 TCs formed during SO, making it the most inactive period since 1979. Although JJA TC activity was enhanced and SO TC activity was suppressed also in some other years like 2002 and 2004 (Fig. 1b,c), 2018 is the most extreme case during the recent forty years. Therefore, in this study we focus on exploring the physical causes of the two periods of extreme WNP TC activity in 2018.

On interannual time scales, the frequency of WNP TCs can be modulated by various climate factors such as the El Niño/La Niña Modoki^1,2^, the Pacific Meridional Mode (PMM)^3–6^, the Atlantic Meridional Mode^7^, tropical North Atlantic (TNA) sea surface temperature (SST)^8–10^, Indian Ocean SST^11–14^, and SST gradient between the southwestern Pacific Ocean and the western Pacific warm pool^15^. A more recent study has further pointed out that regional SST anomalies (SSTA) can modulate TC genesis in different parts of the WNP^16^.

These interannual relationships may change on the inter-decadal time scales. For example, the El Niño Southern Oscillation (ENSO) Modoki has an intensified impact on WNP TC frequency since the early 1990s, which is either attributed to an expansion in area coverage of equatorial central Pacific SST anomalies^17^ or contributed by SST anomalies in several other regions^18^. Indian Ocean SST has an enhanced impact on WNP TC frequency since the late 1970s due to an expansion in area-coverage Indian Ocean SST anomalies^19^. Moreover, TNA SST has a strengthened impact on WNP TC frequency since the late 1970s possibly related to the change in climatological TNA SST^20^.

To recapitulate, previous studies have identified climate modes/drivers that modulate WNP TC frequency on interannual and decadal time scales. Here we aim to assess the climate drivers of the extremely active and inactive TC activity during JJA and SO of 2018, respectively. Different from Wu *et al*.^21^ and Qian *et al.*^22^ who considered the 2018 typhoon season (i.e., June to November or July to November) as an entire active season, the present study is the first attempt to unravel the physical mechanisms underlying two extreme periods in 2018 WNP TC season.

## Data

The 6-hourly TC best-track data are obtained from the China Meteorological Administration - Shanghai Typhoon Institute^23^. We employ the data over the satellite era from 1979 to 2018, during which TC frequency data are more reliable. TCs with at least the tropical storm strength (i.e., maximum sustained wind ≥ 17 m s^–1^) are counted and TC genesis is defined as a TC first reaches the tropical storm strength in this study.

Monthly atmospheric data are obtained from the fifth generation of the European Centre for Medium-Range Weather Forecasts (ECMWF) global reanalysis (ERA5)^24^, we reduce its spatial resolution to 2.5° when performing analyses. The monthly PMM SST index, Niño 3.4 index, Indian Ocean Dipole (IOD) index, and outgoing longwave radiation (OLR) data at the resolution of 2.5°^25^ are obtained from the Physical Sciences Division (PSD) of the National Oceanic and Atmospheric Administration (NOAA)/Earth System Research Laboratory (ESRL). Monthly SST data at the resolution of 1° are acquired from version 4 of the NOAA Extended Reconstructed SST (ERSST)^26^. The ENSO Modoki index (EMI) is acquired from the Japan Agency for Marine-Earth Science and Technology (JAMSTEC).

## Methods

### Calculation of genesis potential index (GPI)

GPI is calculated by^27^

1 $$GPI={|{10}^{5}\eta |}^{3/2}{\left(\frac{H}{50}\right)}^{3}{\left(\frac{{V}_{pot}}{70}\right)}^{3}{(1+0.1{V}_{shear})}^{-2}$$

where *η* is the absolute vorticity at 850 hPa (s^−1^), *Η* is the relative humidity at 600 hPa (%), *V*_*pot*_ is the potential intensity (m s^−1^), and *V*_*shear*_ is the magnitude of vertical wind shear between 850 and 200 hPa (m s^−1^). *V*_*pot*_ can be expressed as^28^

2 $${V}_{pot}^{2}=\frac{{T}_{s}}{{T}_{0}}\frac{{C}_{k}}{{C}_{D}}(CAP{E}^{\ast }-CAPE)$$

where *T*_*s*_ is the SST, *T*_0_ is the TC outflow temperature, *C*_*k*_ is the enthalpy exchange coefficient, *C*_*D*_ is the drag coefficient, the ratio of *C*_*k*_ *to C*_*D*_ is assumed to be 0.9 here. CAPE* and *CAPE* is the convective available potential energy of the ocean surface and the near-surface air near the radius of maximum wind, respectively.

### Numerical model

The National Center for Atmospheric Research (NCAR) Community Atmospheric Model version 5.3 (CAM-5.3)^29^ was used to perform numerical experiments. The model has T31 horizontal resolution (approximately 3.75° × 3.75°) and 26 vertical levels. Parameterization schemes adopted by the model include the deep convection scheme from Zhang and McFarlane^30^, the moist turbulence scheme from Bretherton and Park^31^, the shallow convection scheme from Park and Bretherton^32^, the stratiform cloud microphysics scheme by Morrison and Gettelman^33^, the Rapid Radiative Transfer Method for GCMs (RRTMG) radiation scheme^34^, etc. Details of the experiment design can be found in section 4.2. All the experiments were integrated for 100 years, simulations during the last 80 years were analyzed.

### Significance test

The significance for the anomalies in 2018 is examined by using the Monte Carlo test, which is similar to the bootstrap method used by previous studies^22,35^. The two-tailed Student *t* test is used to examine the significance for difference in the responses between two experiments during 80 years.

## Results

### Observations

**Figure 2:**
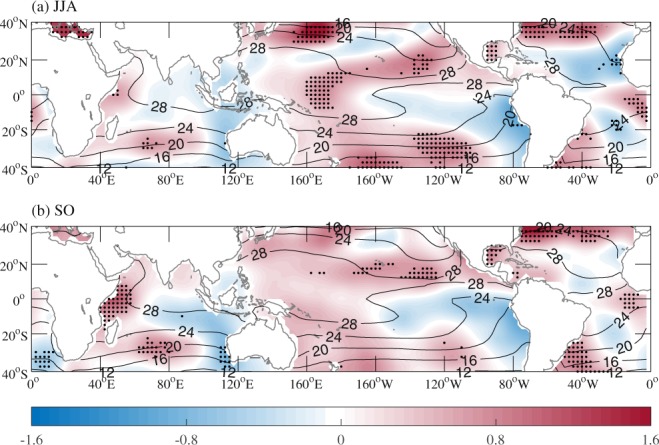
Observed SSTA (shading; °C) during (**a**) JJA and (**b**) SO of 2018 relative to the 1979–2018 climatological mean (contours; °C). Stippled regions indicate SSTAs above the 90% confidence level based on the Monte Carlo test.

During JJA of 2018, the positive PMM and EMI indices exhibited a large magnitude (Fig. 1b), accompanied by the extremely active TC activity in the WNP (Fig. 1a) and warm SSTA in the central Pacific and subtropical eastern Pacific (Fig. 2a). Given the significant impacts of PMM and ENSO Modoki on WNP TC frequency proposed in previous studies^1–3^, the extremely active TC activity during JJA of 2018 was hypothesized to be closely associated with the positive PMM phase and El Niño Modoki. This assumption will be verified and the relative roles of PMM and ENSO Modoki will be quantified using numerical experiments in Section 4.2. However, the positive PMM phase and El Niño Modoki persisted to SO of 2018, albeit with inactive TC activity (Figs. 1a,c and 2b). Questions then arise: why did not PMM and El Niño Modoki modulate WNP TC frequency during SO of 2018 and what suppressed WNP TC activity during SO of 2018?

**Figure 3:**
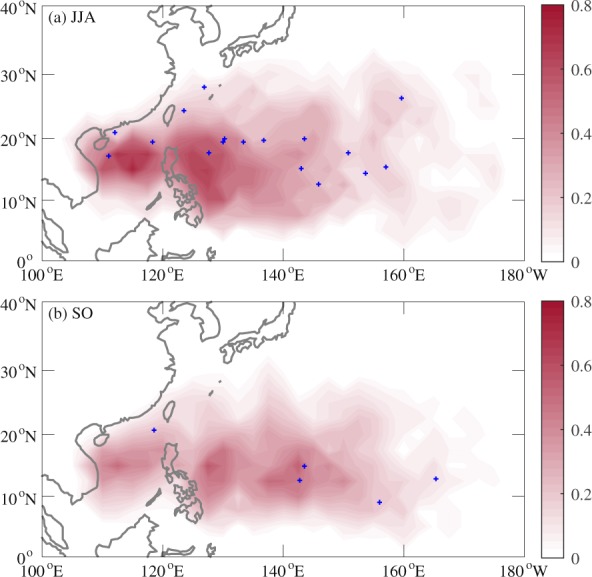
TC genesis locations in 2018 (blue plus signs) and the 1979–2018 climatological mean TC genesis density (shading) during (**a**) JJA and (**b**) SO.

Figure 3 shows TC genesis locations in 2018 and climatological mean genesis density during JJA and SO. Note that two TCs formed at the same location during JJA of 2018. TC formation during JJA of 2018 concentrated in a zonally elongated zone near 20°N over both western and eastern parts of the WNP, which shifted northward compared to the climatological mean (Fig. 3a). During SO of 2018, four TCs formed over the southeastern part of the WNP; however, TC genesis frequency over the SCS and Philippine Sea (PS) was much less than the climatological mean, with only one TC genesis over the SCS and no TC genesis over the PS (Fig. 3b), which was responsible for the extremely inactive period of TC activity in the WNP.

**Figure 4:**
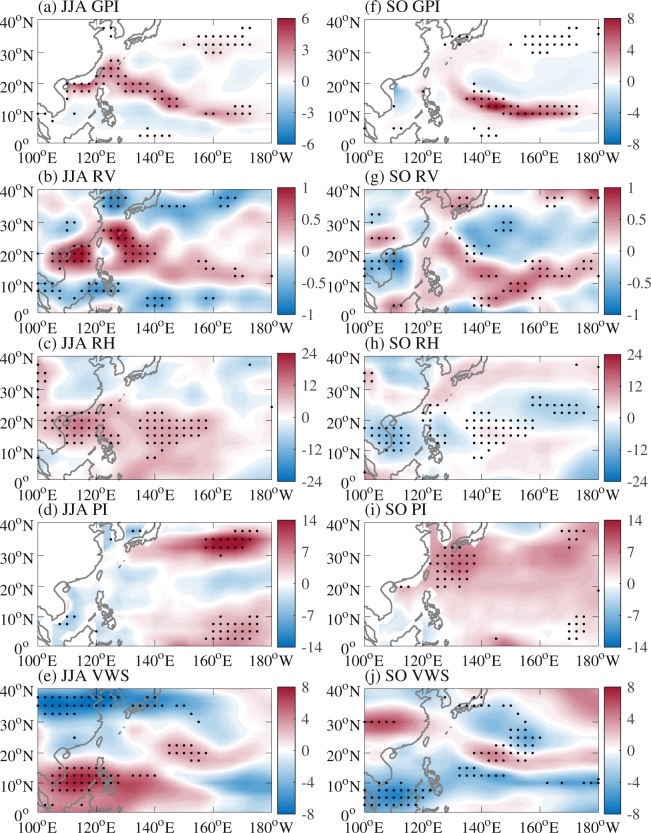
Observed anomalies of (**a**) GPI, (**b**) 850-hPa relative vorticity (10^−6^ s^−1^), (**c**) 600-hPa relative humidity (%), (**d**) potential intensity (m s^−1^), and (**e**) 850–200-hPa vertical wind shear (m s^−1^) during JJA of 2018, and (**f**) GPI, (**g**) 850-hPa relative vorticity (10^−6^ s^−1^), (**h**) 600-hPa relative humidity (%), (**i**) potential intensity (m s^−1^), and (**j**) 850–200-hPa vertical wind shear (m s^−1^) during SO of 2018 relative to the 1979–2018 climatological mean. Stippled regions indicate values above the 90% confidence level based on the Monte Carlo test.

To examine the important factors responsible for the extremely enhanced and suppressed TC genesis frequency particularly over the main development region of the WNP during JJA and SO of 2018, respectively, we adopt the widely used GPI. Figure 4 shows the anomalies of GPI and its four components during JJA and SO of 2018 compared to their respective climatological means. The approximately dipole pattern in the tropical WNP with positive GPI anomalies in the north and negative GPI anomalies in the south during JJA of 2018 (Fig. 4a) was consistent with the northward shift of TC formation (Fig. 3a). During SO of 2018, negative GPI anomalies in the SCS and PS (Fig. 4f) were in line with the extremely low TC genesis frequency there (Fig. 3b); and positive GPI anomalies in the southeastern part of the WNP (Fig. 4f) corresponded to the region with four TCs (Fig. 3b). This indicates that GPI can well represent TC formation during both JJA and SO of 2018. Among the four components of GPI, only the pattern of low-level relative vorticity was highly consistent with that of GPI, low-level relative vorticity (Fig. 4b,g) was therefore the main contributor to the anomalous TC formation during both JJA and SO of 2018.

**Figure 5:**
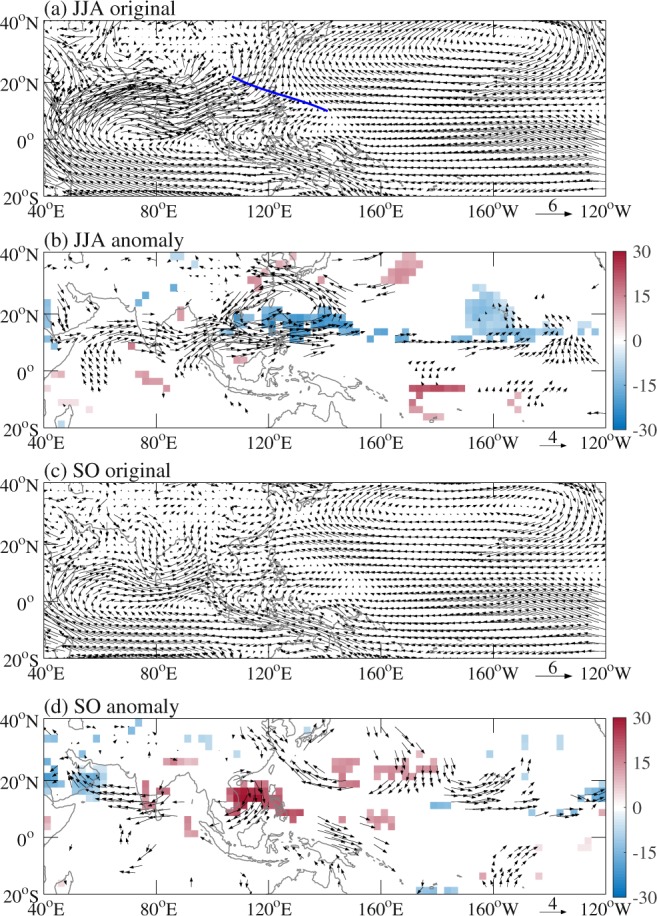
Observed 850-hPa wind (vector; m s^−1^) during (**a**) JJA and (**c**) SO of 2018 and anomalies of 850-hPa wind (vector; m s^−1^) and OLR (shading; W m^−2^) during (**b**) JJA and (**d**) SO of 2018 relative to the 1979–2018 climatological mean. Thick blue line in (**a**) indicates the monsoon trough line. Signals shown in (**b**) and (**d**) are significant above the 90% confidence level based on the Monte Carlo test.

Figure 5 shows 850-hPa winds as well as the anomalies of 850-hPa winds and OLR during JJA and SO of 2018. Climatological mean 850-hPa winds are also calculated for comparison (Figures not shown). During JJA of 2018, positive low-level relative vorticity anomalies (Fig. 4b) were associated with enhanced convection and anomalous low-level cyclonic circulation over the WNP (Fig. 5b), which coincided with an eastward extension of the monsoon trough (Fig. 5a), similar to Wu *et al*.^21^. The enhanced monsoon trough can provide favorable conditions and interact with intraseasonal oscillations and synoptic-sale disturbances and hence increase TC genesis frequency^21,36–38^. During SO of 2018, negative low-level relative vorticity anomalies over the SCS and PS (Fig. 4g) were accompanied by suppressed convection and anomalous low-level anticyclonic circulation (Fig. 5d), which coincided with a weaker-than-normal monsoon gyre (Fig. 5c), responsible for few TC formations over the SCS and PS. This was in line with weak intraseasonal and synoptic-sale signals during SO (especially October) of 2018 shown in Wu *et al.*^21^ (see their Fig. 11).

### Numerical experiments

What caused the above-mentioned anomalous large-scale circulation responsible for the extreme TC activity during JJA and SO of 2018? The anomalous large-scale circulation during JJA may be attributed to SST anomaly associated with the positive PMM phase and El Niño Modoki. However, the positive PMM phase and El Niño Modoki persisted from JJA to SO. Why was the anomalous large-scale circulation over the SCS and PS during SO different from that during JJA? We speculate that the tropical Indian Ocean SSTA associated with the positive IOD phase (Fig. 2b) may induce the anomalous anticyclonic circulation over the SCS and PS during SO. These assumptions will be verified with numerical experiments.

**Figure 6:**
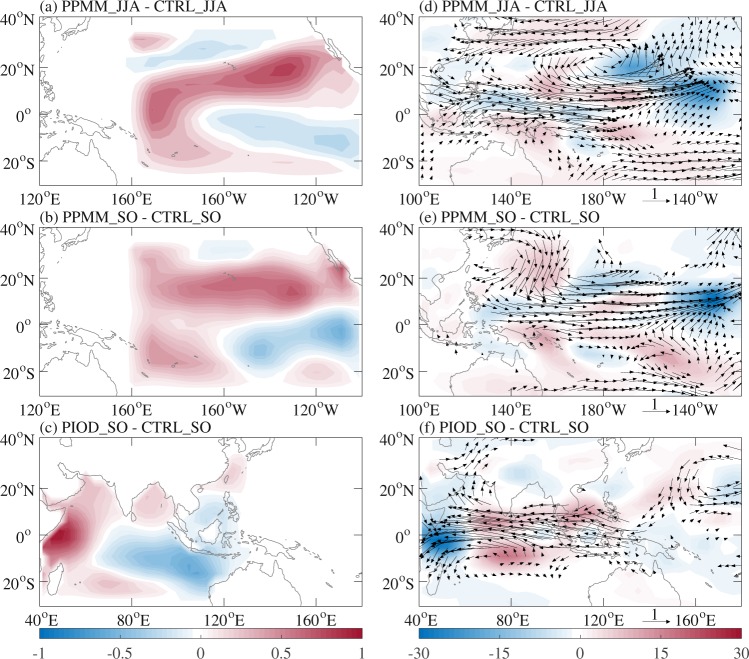
Differences in prescribed SST (°C) (**a**) between the PPMM_JJA and CTRL_JJA experiments, (**b**) between the PPMM_SO and CTRL_SO experiments, and (**c**) between the PIOD_SO and CTRL_SO experiments with the CAM-5.3, as well as differences in 850-hPa wind (m s^−1^; vector) and OLR (W m^−2^; shaded) (**d**) during JJA between the PPMM_JJA and CTRL_JJA experiments, (**e**) during SO between the PPMM_SO and CTRL_SO experiments, and (**f**) during SO between the PIOD_SO and CTRL_SO experiments. Signals shown in (d)–(f) are significant above the 90% confidence level based on the two-tailed Student *t* test.

We first performed two sets of numerical experiments using CAM-5.3. The first set includes two experiments, one was prescribed with the observed climatological SST during JJA (CTRL_JJA experiment), and the other with SSTA during JJA of 2018 in the PMM region that includes the region for ENSO Modoki (Fig. 6a) added to the observed climatological SST during JJA and the same as the CTRL_JJA experiment elsewhere (PPMM_JJA experiment). The second set includes three experiments, one is prescribed with the observed climatological SST during SO (CTRL_SO experiment), and the other two with SSTA during SO of 2018 in the PMM/IOD region (Fig. 6b,c) added to the observed climatological SST during SO and the same as the CTRL_SO experiment elsewhere (PPMM_SO/PIOD_SO experiment). The differences between PPMM_JJA and CTRL_JJA experiments (PPMM_JJA minus CTRL_JJA) and between PPMM_SO/PIOD_SO and CTRL_SO experiments (PPMM_SO/PIOD_SO minus CTRL_SO) are used to represent the changes forced by SSTA in different regions.

During JJA of 2018, the differences in 850-hPa wind and OLR between the PPMM_JJA and CTRL_JJA experiments (Fig. 6d) exhibited cyclonic circulation anomalies and enhanced convection over the WNP, which highly resembled the observations (Fig. 5b), suggesting the important role of the heating associated with El Niño Modoki and the positive PMM phase in modulating the anomalous low-level cyclonic circulation through a Gill-type Rossby wave response^37^. During SO of 2018, comparison between the simulations (Fig. 6e,f) and observations (Fig. 5d) indicated that the equatorial heating associated with the positive IOD phase induces anomalous low-level anticyclonic circulation over the North Indian Ocean, SCS and PS (Fig. 6f) through a Gill-type Kelvin wave response^39^, and the off-equatorial heating associated with the positive PMM phase induces anomalous low-level cyclonic circulation over the central and eastern North Pacific (Fig. 6e) through a Gill-type Rossby wave response^39^. Therefore, the numerical experiments demonstrated that the positive PMM phase and El Niño Modoki were the crucial factors responsible for the extremely active WNP TC activity during JJA of 2018 and the positive IOD phase was the key factor responsible for the extremely inactive WNP TC activity during SO of 2018. The situation during SO of 2018 turned out to be exceptional from the statistical relationship between WNP TC activity and IOD.

**Figure 7:**
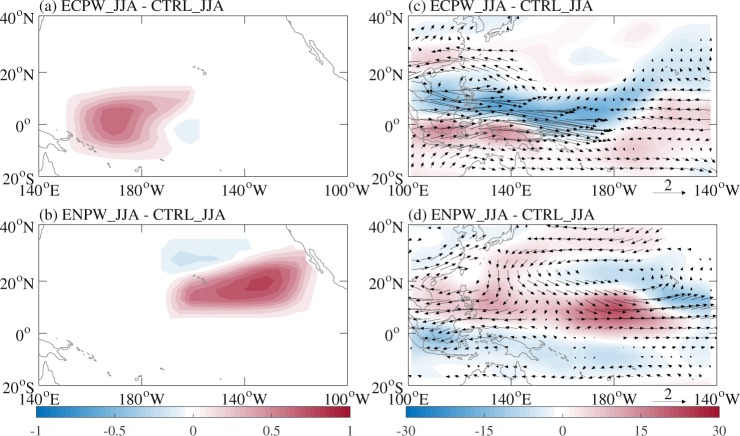
Differences in prescribed SST (°C) (**a**) between the ECPW_JJA and CTRL_JJA experiments, and (**b**) between the ENPW_JJA and CTRL_JJA experiments with the CAM-5.3, as well as differences in 850-hPa wind (m s^−1^; vector) and OLR (W m^−2^; shaded) during JJA (**c**) between the ECPW_JJA and CTRL_JJA experiments, and (**d**) between the ENPW_JJA and CTRL_JJA experiments. Signals shown in (**c**) and (**d**) are significant above the 90% confidence level based on the two-tailed Student *t* test.

Two additional experiments were performed to investigate the relative role of the positive PMM phase and El Niño Modoki in enhancing WNP TC frequency during JJA of 2018. One was prescribed with SSTA during JJA of 2018 in the equatorial Central Pacific (Fig. 7a, representative of El Niño Modoki) added to the observed climatological SST during JJA and the same as the CTRL_JJA experiment elsewhere (ECPW_JJA experiment), and the other with SSTA during JJA of 2018 in the subtropical eastern North Pacific (Fig. 7b, representative of the positive PMM phase) added to the observed climatological SST during JJA and the same as the CTRL_JJA experiment elsewhere (ENPW_JJA experiment). Through comparison with the observations in Fig. 5b, the forced 850-hPa wind and OLR suggested that El Niño Modoki was the key factor for the anomalous low-level cyclonic circulation over the entire WNP (Fig. 7c) and the positive PMM phase was only responsible for the anomalous low-level cyclonic circulation over the eastern part of the WNP (Fig. 7d). This demonstrated that El Niño Modoki (the positive PMM phase) may play the primary (secondary) role in the extremely active WNP TC activity during JJA of 2018. This finding is generally consistent with Wu *et al*.^21^. Note that the simulated responses to El Niño Modoki (Fig. 7c) do not fully coincide with the observed variations in Wu *et al*.^2^ (their Fig. 8b), and this may be because their observed composites could contain signals from other concurrent climate factors such as the subtropical eastern North Pacific warming (their Fig. 3b).

## Conclusion

The 2018 WNP TC season exhibited extremely active TC activity during JJA and extremely inactive TC activity during SO. Through observational analyses and numerical experiments, this study has found that different climate patterns were responsible for the extreme WNP TC activity in 2018. During JJA of 2018, the anomalous cyclonic circulation over the WNP, which was favorable for extremely high TC genesis frequency and northward-shifted TC genesis locations, was induced by the heating associated primarily with El Niño Modoki and secondarily with the positive PMM phase. During SO of 2018, the anomalous anticyclonic circulation over the North Indian Ocean, SCS and PS, which resulted in extremely low TC genesis frequency over the SCS and PS (and thus extremely low TC genesis frequency over the entire WNP), was induced by the equatorial heating associated with the positive IOD phase. The results imply that SSTA in different regions should be properly considered in seasonal prediction of the WNP TC frequency and individual prediction models for subregions of the WNP would be beneficial to improving seasonal prediction of TC frequency over the entire WNP.

## Data Availability

The NCEP/NCAR reanalysis, ERSST V4, and OLR data, and the PMM, IOD, and Niño 3.4 indices are provided by the U.S. NOAA/OAR/ESRL PSD from their website at https://www.esrl.noaa.gov/psd/. The ENSO Modoki index is provided by the JAMSTEC at http://www.jamstec.go.jp/frsgc/research/d1/iod/DATA/emi.monthly.txt. TC best track dataset is provided by the China Meteorological Administration - Shanghai Typhoon Institute at http://tcdata.typhoon.org.cn/en/.
